# Effectiveness of a delegated primary care model in rural areas: design and methodology of a longitudinal observational study

**DOI:** 10.1186/s12913-025-12742-5

**Published:** 2025-04-18

**Authors:** Julia Bräuer, Romina Lörzing, Eckhard Nagel, Martin Emmert, Reiner Hofmann

**Affiliations:** 1 Institute for Management in Medicine and Health Sciences, Project Office of the Medical Campus Upper Franconia, University of Bayreuth, Bayreuth, Germany; 2 Institute for Management in Medicine and Health Sciences, University of Bayreuth, Bayreuth, Germany; 3 Healthcare Management and Health Services Research, University of Bayreuth, Bayreuth, Germany

**Keywords:** Delegation, Effectiveness, General practitioners, Healthcare professionals, Home visits, Primary care, Rural healthcare

## Abstract

**Background:**

The shortage of general practitioners (GPs) in Germany, particularly in rural areas, is a pressing issue that requires innovative and sustainable solutions. One promising approach is the integration of trained medical assistants to support GPs by handling specific tasks, thus enhancing the efficiency of healthcare delivery. Delegated home visits can be carried out by healthcare professionals (HPs) with a well-defined range of tasks.

**Methods:**

This quasi-experimental, non-randomised longitudinal observational study runs from January 2023 to June 2025. The study participants are patients cared for by participating GPs, selected based on defined inclusion criteria, who receive regularly delegated home visits. Health-related quality of life (HRQoL) and satisfaction is assessed at three data collection points (t0 as baseline before starting the intervention, t1 after the first testing and data collection phase, and t2 after the second data collection phase). For assessing the HRQoL, we use the Short-Form-Health Survey (SF-12), and satisfaction is being measured using the patient satisfaction questionnaire ZUF-8. These parameters, as well as the continuously collected parameters (kilometers driven, travel and setup time), are analysed statistically (descriptive statistics, paired t test, Wilcoxon test, ANOVA). At t1 and t2, focus group interviews are conducted with the assistants on their satisfaction. They allow to identify the potential for concept improvement by qualitative content analysis.

**Discussion:**

The “VERSORGT am ORT” (VaO, engl. “PROVIDED on SITE”) project aims to optimise healthcare by using scarce resources in a more efficient way, maintaining patient-professional trust, and extending delegation. It seeks to implement care plans with minimal location changes and generate real-world evidence. Due to geographical and policy limitations, the study will have comparatively few participants and will use a repeated measures design instead of a control group to assess patient HRQoL and satisfaction over time. Changes in HRQoL cannot be directly linked to the new care model due to unclear causalities. Mergenthal et al. reported that 59% of GPs in rural areas delegate home visits to healthcare professionals, saving time. The VaO project aims to further relieve GPs, increasing efficiency without compromising care quality.

**Trial registration:**

DRKS00033915 – Registration Date 19/03/2024.

**Supplementary Information:**

The online version contains supplementary material available at 10.1186/s12913-025-12742-5.

## Background

One of the stated objectives of the EU Member States is to ensure access to modern and efficient healthcare for all [1]. However, Germany faces challenges to providing regionally equitable healthcare. Rural areas, in particular, are at risk of reduced medical services, with many patients experiencing dwindling medical resources. As demographic shifts and rising life expectancy lead to an increasing number of older patients with multiple chronic conditions, the availability of medical care continues to decline. This growing demand necessitates innovative approaches to healthcare delivery [2].

Germany ranks in the upper third of the 32 Organisation for Economic Cooperation and Development (OECD) countries in terms of general practitioner density [3]. In 2023, the absolute number of GPs practicing in Germany was 55,127 [4], representing 36% of ll 153,726 SHI-accredited physicians [5]. However, their distribution is uneven, resulting in inadequate primary care in some regions. Demographic change will exacerbate this situation, with 11.9% of themedical workforce aged over 65 by 2022 [6], leading to an anticipated shortfall of 10,851 GP posts that will remain unfilled in 2035 [7]. Overall, there is an urgent need to promote young talent to take over vacant doctor offices, as the age-related retirement of many GPs is being offset by an acute shortage of young talent [8].

A semiannual audit of the Bavarian Association of Statutory Health Insurance Physicians (Kassenärztliche Vereinigung Bayern, KVB) indicated that rural healthcare in Bavaria, particularly in the field of GPs, is at risk in many regions [9]. This is where the innovative VaO care model comes in and analyses the structures of existing delegation concepts in order to organise the care of home-visit patients in a more efficient way. The almost 25,000 inhabitants of our examined example region, the Streutal Valley in Northern Bavaria, are already facing this situation mentioned above. The structure of the eleven regional GPs with an average age of 66.8 years facing an average retirement age in Germany of 64.4 years in 2022 suggests that some of them will retire in the coming years [10, 11]. A study by the University of Trier shows the resulting difficulty: 36% of all human medicine doctors in Germany are GPs [5] but only 12% of the students tend to this subject [12].

Several delegation concepts exist and aim to reduce the workload of GPs, such as different types of specialised care assistants, ‘care assistants in the GP practice’ (VERAH), ‘nonphysician practice assistants’ (NäPA) or ‘physician-supported, community-based, e-health-supported, systemic intervention’ (AGnES)- hereafter referred to as healthcare professionals (HPs). These specially trained HPs can carry out defined activities delegated by GPs, including home visits. This frees up GP time for patient care, especially in rural areas with long distances to and between patients’ homes [13].

The delegation of GPs’ activities is therefore a promising strategy to address care gaps. However, the availability of delegation recipients is also finite. To maximize the efficiency of healthcare services, it is crucial to optimise the organization of service provision. The VaO concept addresses this by relocating home visits to nearby, well-equipped VaO rooms for patients who are physically able to access them. These rooms serve as treatment centers used exclusively by healthcare professionals (HPs) and patients with GP recommendations. Within the VaO care model, the scope of services and the HPs delivering them stay as they are established, medical management remains under GP supervision. Services provided include measuring blood sugar levels, changing dressings, or taking blood samples, as defined by the Agreement on the delegation of medical services to nonmedical staff in outpatient care of contract physicians care in accordance with § 28 Para. 1 S. 3 SGB V by the Association of Statutory Health Insurance Physicians in cooperation with the National Association of Statutory Health Insurance Funds [14].

## Objectives

The overall aim is to (1) implement the VaO concept in a real-world setting in the Streutal Valley in Northern Bavaria and to (2) evaluate its effectiveness by assessing economic efficiency, satisfaction and HRQoL in compared to the usual care provision.

Therefore, the primary objective is to conduct a health economic evaluation of VaO as a minor modification and resource-saving interdisciplinary form of care to mitigate the shortage of GPs in rural areas. The secondary objective is to test its feasibility and integration into the daily routines of patients, HPs and GPs as well as to evaluate the psychosocial effects of the concept. The following hypotheses are being tested to answer the selected research question.

- Hypothesis 1: The monetary expenses and savings of the VaO care model lead to economically neutral to positive results for the GPs running the rooms.
- Hypothesis 2: The VaO care model reduces average working time spent in nonpatient areas (travel and setup time).
- Hypothesis 3: The VaO concept does not lead to a change in patients’ HRQoL.
- Hypothesis 4: The VaO concept does not lead to a change in patients’ and HPs satisfaction.

### Trial design

The study is designed as a non-randomised, single-arm longitudinal observational study with repeated measurement points in the context of health services research. It is conducted from January 2023 to June 2025, spanning a total duration of 30 months and comprising five phases. The first one, the preparatory phase focused on the requirements of implementation. These were assessed by participating GPs and selected according to the following criteria: (1) village where home visits are carried out (2), number of patients receiving home visits, and (3) share suitable for visiting a VaO room. Suitable premises were selected in collaboration with local mayors. A checklist in accordance with the Association of General Practitioners in Germany (VirchowBund) was used to ensure alignment of the facilities [15]. The criteria included, for example, accessibility, sanitary facilities and stable internet connections. The rooms were inspected by HPs and GPs.

In the initial assessment and testing phase, patients’ care in VaO rooms by their known HPs starts. The HPs continuously monitor the patients’ state of health to determine their suitability for care in VaO rooms [16]. The subsequent interim evaluation and concept adjustment phase aims to correct weaknesses, sources of risk and errors from the initial assessment and testing phase by conducting a focus group interview with the participating HPs on their satisfaction with the use of the room. Sources of risk and errors are targeted while adjustments to the VaO concept optimise processes. In the second survey phase, the time and distance parameters of patient care are continuously collected. A final evaluation is carried out at the end of the study to test the hypotheses.

### Study setting

The study is being conducted in VaO rooms, which have been specially set up for this purpose. The rooms are furnished and aligned in accordance with criteria of the VirchowBund for medical practices [15]. There are seven VaO rooms located in the villages of Stockheim, Oberstreu, Hendungen, Hausen, Oberwaldbehrungen, Unsleben and Bastheim in the bavarian region Streutal. In the initial assessment and testing phase, those are used and shared by three practices, of which two are joint practices.

### Patient recruitment

Patients are recruited directly by the participating GPs. Patients who meet the following inclusion criteria are contacted directly by their GPs and provided with further information about the study. The final date for patient enrolment is December 31, 2024. Data on travel time, setup time and distance will be collected up to this date. Data on HRQoL and satisfaction will be subjected to analysis for all patients enrolled up to July 31, 2024, in order to be able to map the development over half a year (from t0 to t1).

### Eligibility criteria

We defined eligibility criteria for all participating groups: GPs, HPs and patients. First, GPs qualify for participation if they are established in the Streutal region. Second, HPs qualify if they are employed by a participating GP and care for home visit patients. Third, patients qualify for participation if they are selected by the participating GPs based on the following defined inclusion criteria:

- Patient is treated by a participating GP.
- Patient receives home visits from a HP that is employed by a participating GP.
- Patient provides voluntary and written consent for pseudonymized recording of HRQoL and satisfaction as well as processing to and analysing by the evaluating team.
- Patient provides voluntary and written consent for anonymised recording of the care provided and the structural data (distance and time) of the home visit route as well as processing to and analysing by the evaluating team.
- Patient is capable of visiting the VaO room (according to the assessment of the GP in charge).
- Patient is 18 years of age or older.
- Patient has sufficient knowledge of German.

The patients participating in the study provide written informed consent to their GPs after being informed of the details of the study and their participation, particularly the associated change in their care (the establishment of the VaO room, where basic treatment sequences may be conducted by the HPs). They are also informed of their voluntariness and the right to withdraw at any time, which entails a change to the previous way of care through home visits.

### Intervention description

After being selected by the participating GPs and providing informed consent, patients receive their medical care through delegated services in one of the established VaO rooms during all phases of the study as long as they meet the inclusion criteria. They receive the same care as if they were at home – the range of services provided by HPs is not extended, only the location of the service changes. For instance, services such as the collection of blood samples, the measurement of blood sugar levels or the treatment of wounds may be provided at the VaO rooms. The HP-patient relationship remains the same as well, as the HPs continue to see the same patients they have seen in their home visits.

The use of the VaO rooms is organised independently of the practices. This means that presence times and patient timing are managed by the HPs themselves so that the VaO rooms can be individually integrated into practices’ daily routine. For example, the frequency of opening the VaO room or the type of patient appointment can be determined by the practices. This concept is based on continuity, as the combination of GP, HP, patients and services remains unchanged. This makes it possible to change the location of care provision without interfaces and without loss of any information.

### Outcomes

The primary outcomes include time saved in the nonpatient area: travel and setup time. Based on the patients who are cared for and the services that are given in the VaO rooms (VaO scenario), the amount of travel and setup time can be analysed and compared to the time that would have been needed if services were given in the original home visiting route (home visit scenario).

**Fig. 1:**
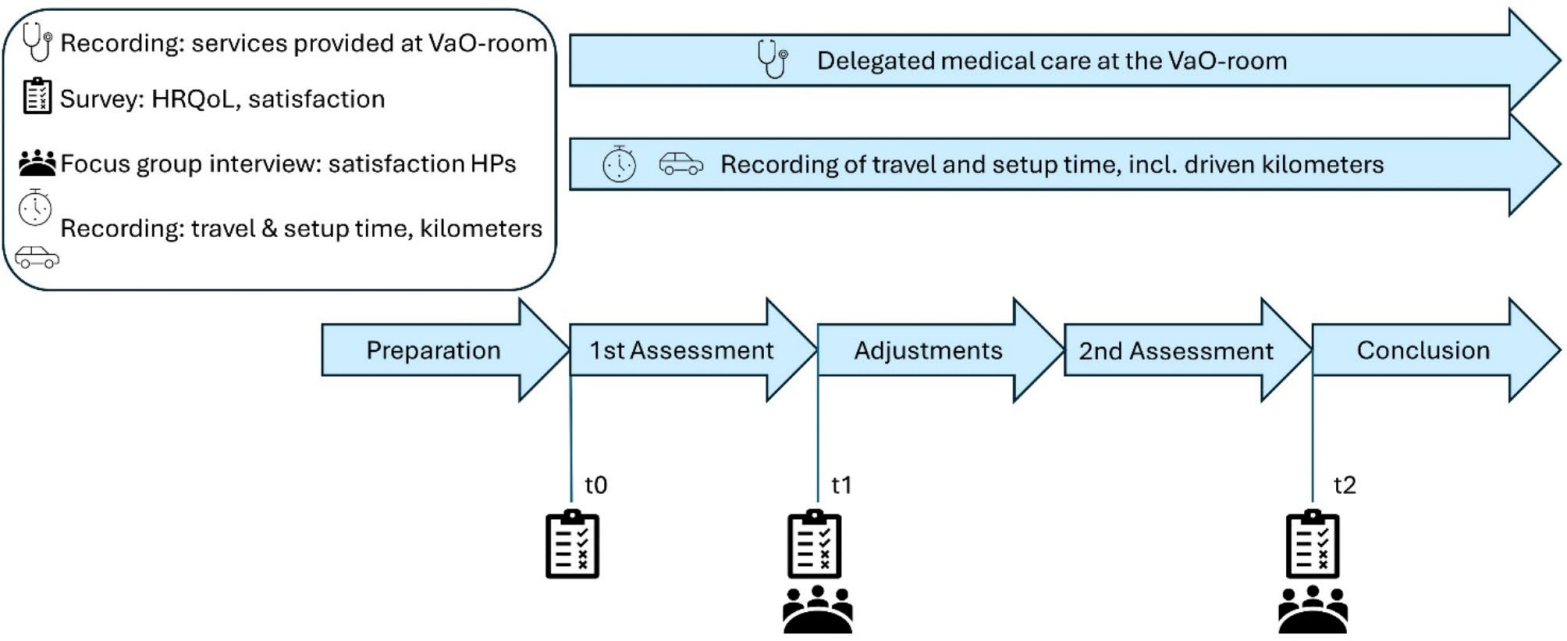
Survey dates of HRQoL, satisfaction, time and distance

Two secondary outcomes are considered. First, patients’ HRQoL is assessed using the SF- 12 questionnaire which is based on a physical and psychological summated scale. The standardised and validated questionnaire is used because it is known to be simple and quick to administer in the assessment of HRQoL. As a generic measurement instrument for self-reported HRQoL it is particularly suitable for the target group, as it consists of older patients who are asked to make independent statements about their health [17]. The questionnaire is comparatively easy to use as it consists of only 12 items [18]. Second, patient satisfaction is measured using the ZUF- 8 instrument, which is a standardised tool for measuring satisfaction in an inpatient context. It was selected on the basis of its multiple uses in studies and appropriate evaluation of the quality criteria [17, 19]. To examine the changes in HRQoL and patient satisfaction, these parameters are surveyed at three time points during the study, as shown in Fig. 1 (time points t0 as baseline, t1 after six months as midterm, and t2 after 12 months as final examination).

### Sample size

As the study initially pursues the general proof of concept and has an observational character, it aims to explore complex interactions and the evolution of the use of VaO rooms rather than to quantify specific instances. We see the development of the usage of the VaO rooms, the number of patients receiving care and the given services as indicators of study success. The sample size estimation is conducted using the tool G*Power Version 3.1.9.7 of Heinrich Heine University of Düsseldorf. The calculation is based on an expected effect size of 0.25, an alpha error level of 0.05 and a statistical power of 0.80. Given the repeated measures ANOVA design, the number of groups is set to one with three measurement time points. Additionally, a correlation of 0.5 between repeated measures is assumed, and the nonsphericity correction is set to the default value of one. This results in a required total sample size of 28, with an actual power of 0.81.

### Data collection and management

#### Plans for assessment and collection of outcomes

##### Patient satisfaction and HRQoL

The participating patients are administered the SF- 12, a questionnaire frequently used in clinical studies, to assess their HRQoL. Additionally, the ZUF- 8, a questionnaire designed to assess patient satisfaction, is used after being adapted to the context of the “home visit” or “VaO” setting. Both questionnaires are completed at three points in time (t0 before the first VaO room visit of the patient, t1 after six months, t2 after 12 months) during the course of the study to observe and evaluate changes.

In addition, focus group interviews will be conducted with the participating HPs on their satisfaction with VaO concept at survey times t1 and t2. Therefore, we designed an interview guideline which covers important objects like time saving or the changes due to the use of VaO in everyday working life (Appendix A). The interviews are intended to capture the range of opinions of the HPs regarding the innovative VaO concept in an open discussion to validate or question its components. Additionally, potential success factors or barriers to the implementation of the concept will be identified.

##### Time and distance savings

As part of the initial data collection and testing phase, distances travelled are continuously monitored and collected for two scenarios: the VaO scenario, in which all eligible patients are cared for in VaO rooms; and the home visit scenario, in which all patients are cared for at home. The online route planner MapQuest is used to calculate the optimal routes for each of the scenarios, with the results presented in the form of distance and time estimates [20]. In addition to the abovementioned measures of time spent away from the patients, the setup times (preparation and follow-up of materials) for services performed during the home visit were measured before the beginning of the study with the participating HPs. These setup times are also included in the evaluation of time savings.

##### Costs

The costs of the innovative form of care are calculated using the existing remuneration models for medical assistants in job group IV of the German Collective Labour Agreement for Medical Assistants. The average salary and the average monthly working time of a full-time HP are calculated using the collective agreement for medical assistants of the Bavarian Medical Association [21, 22]. The exact values are one basis for calculating the expected cost savings from VaO.

To measure the cost-effectiveness of the intervention, the incremental cost-effectiveness ratio (ICER) of the innovative care system VaO in comparison to the standard method of care is calculated. Mathematically, the ICER sets the difference in costs in relation to the difference in benefits and allows to illustrate the drivers and limitations (e.g., effects of VaO room utilisation, logistical challenges) of cost-effectiveness:

$$\mathrm{ICER}\;=\;({\mathrm C}_{\mathrm{VaO}}-{\mathrm C}_{\mathrm{standard}})/({\mathrm B}_{\mathrm{VaO}}-{\mathrm B}_{\mathrm{standard}})$$

C_VaO_ includes the calculated costs of the care system VaO, namely, room rent (including maintenance and cleaning), which is calculated from the local standard comparative rent, as well as the costs of travel distances and times according to the VaO route planning when using the VaO rooms.

C_standard_ includes the cost of travel distances and times according to standard route planning.

B_VaO_ includes the benefit expressing change in satisfaction and HRQoL from t0 to t1 and t2 as mentioned above as a reflection of the satisfaction with the innovative form of care.

B_standard_ includes the benefit expressing average HP and patient satisfaction at time t0, reflecting satisfaction with their usual form of care.

#### Data management

All data get pseudonymized or anonymised by GPs or HPs before they reach the University of Bayreuth and are stored in secure areas. The processing of pseudonymized data from the questionnaires and anonymised transcripts of focus group interviews at the University of Bayreuth is conducted by research assistants who signed a written declaration of confidentiality. Recording of paper documents in separate temporary work files is noted individually, while the objective of double entry with subsequent plausibility checks is to prevent or identify input errors. Once data have been merged into a single, error-corrected working file, this serves as basis for the scientific evaluations. The temporary work files are deleted at this point, the transcripts and paper documents are archived, stored securely and inaccessible to third parties until the end of the evaluation. Only the working file will be stored for 15 years after study ending while all other data will be deleted after the final project report is accepted by the sponsor.

It is explicitly stated that third parties not directly or indirectly involved in the collection or analysis of data are not be granted access to the study data. The data analysis is conducted exclusively by named, contractually employed staff of the VaO project at the University of Bayreuth. All employees are trained in accordance with the data protection guidelines and are contractually obliged to comply with them. No data will be disseminated beyond the results of the evaluations. The results will be provided in an aggregated way with fully anonymised quotations, and do not permit any inferences to be drawn about individual patients.

#### Confidentiality

All data collected for the purposes of the study are confidential and will not be disclosed to third parties. To safeguard any personal data of the participants, all data collected are pseudonymized or anonymised. Pseudonym lists are retained by GPs who provided the information, as are the patient consent forms. As no medical information is required for the study, only the date of birth and sex of the participants are collected. Prior to the commencement of the study, the team received ethical approval from the Ethics Committee of the University of Bayreuth. This approval was granted following the submission of the study protocol including consent forms and questionnaires for assessment.

### Statistical methods

#### Statistical methods for primary and secondary outcomes

Table 1 shows the statistical methods used to analyse quantitative data.

**Table 1:** Statistical methods used for quantitative data sets

Data	Points in time	Statistical method
SF- 12	t0, t1, t2	Descriptive statistics, paired t test, ANOVA with repeated measures
ZUF- 8	t0, t1, t2	Descriptive statistics, paired t test, ANOVA with repeated measures
Kilometers	continuous	Descriptive statistics, paired t test, Wilcoxon test
Time	continuous	Descriptive statistics, paired t test, Wilcoxon test

The baseline data (t0) on HRQoL and patient satisfaction are analysed using descriptive statistics. To assess the changes that occurred after the first survey and testing phase, data collected at t0 and t1 are compared using paired t-tests or Wilcoxon test as part of an interim analysis. Upon completion of the second survey and testing phase, all data from the three measurement points (t0, t1 and t2) are subjected to a repeated measures ANOVA to compare outcomes across all three time points.

Additionally, the distance travelled and time spent are compared between the VaO scenario and the home visit scenario. Given the conditions shown, paired t tests or Wilcoxon test are used to determine whether there are any discernible differences in the parameters under examination.

The emphasis is on understanding underlying trends, patterns and on gaining real world evidence.

### Dissemination plans

The results of the study will be published in open access peer-reviewed journals. To maximise the impact of the project and to support the dissemination of its results including knowledge transfer with the scientific community, the scientific results will be published in high-ranked journals and presented at considerable congresses. Furthermore, relevant stakeholders including researchers, practice owners, healthcare professionals and policy makers, will be informed directly through appropriate channels.

## Discussion

VaO addresses several healthcare challenges through its straightforward design and use of existing resources. The study aimes to explore the effective use of scarce human resources, to maintain the relationship between GPs, HPs and patients, and to extend the concept of delegation. The implementation of the care plan achieves its objectives with minimal changes to the location of service provision while generating real-world evidence within the context of everyday care.

Due to the geographical limitations of the project region and the strict health policy criteria regarding home visits, the study is expected to have a relatively small number of participants. A control group was omitted since the development of HRQoL and patient satisfaction across the three observation points uses a repeated measures design. While the first measurement t0 is placed at the baseline before starting the intervention, t1 and t2 show the development after six resp. twelve months of testing and data collecting.

This design is considered appropriate for evaluating the form of care. Repeated measurements enable the assessment of long-term effects [23, 24]. Moreover, comparing the two scenarios before and after implementation allows for the identification and discussion of any observed improvements or deteriorations. However, it is not possible to directly attribute the observed changes in HRQoL to the implementation of the new care concept due to uncertainties about causalities [25].

A study by Mergenthal et al. revealed that 59% of GPs surveyed had their home visits carried out exclusively by HPs. The study also revealed that HPs are often employed in rural areas allowing the GPs to save a significant amount of time [26]. In combination with VaO and the expected savings in working time in the nonpatient area, GPs could be relieved to a greater extent. The authors see the most significant advantage of the project in this enhanced efficiency. Given that the spatial alterations are minimal, the actual care remains unaltered, and there is no expected loss of quality.

### Trial status

Version 1, status 31/03/2023. Recruitment began at 22/05/2023 and is still ongoing until 31/12/2024. Trial was registered at the German Clinical Trials Register (DRKS00033915) – Registration Date 19/03/2024.

## Supplementary Information

Supplementary Material 1.

## Data Availability

No datasets were generated or analysed during the current study.

## Abbreviations

AGnES: Physician-relieving, community-based, e-health-supported, systemic intervention (Arztentlastende, Gemeindenahe, E-Health-gestützte, Systemische Intervention)
DMC: Data monitoring committee
GP: General practitioner
HP: Healthcare professional
HRQoL: Health-related quality of life
ICER: Incremental cost effectiveness ratio
KVB: Bavarian Association of Health Insurance Doctors (Kassenärztliche Vereinigung Bayern)
NäPa: Nonphysician practice assistants (Nichtärztliche Praxisassistenz)
OECD: Organisation for Economic Co-operation and Development
SF-12: Short-Form 12
VaO: VERSORGT am ORT (engl. “PROVIDED on SITE”)
VERAH: Care assistants in the GP practice (Versorgungsassistenz in der Hausarztpraxis)
