# Supplementary material for: Effectiveness of a delegated primary care model in rural areas: design and methodology of a longitudinal observational study
Source: BMC Health Serv Res. 2025 Apr 18;25:567. doi: 10.1186/s12913-025-12742-5 (PMC12007290; doi:10.1186/s12913-025-12742-5)
Supplement: Supplementary file 1 — Supplementary Material 1. [file 12913_2025_12742_MOESM1_ESM.pdf]

Introduction, information on the project, confidentiality

- *In the course of the interview, I will ask you various (open) questions, for which I will basically ask you to just tell me everything that comes to your mind.*

- *Please take your time answering the questions and don't feel pressured.*

- *I will not interrupt you while you are answering. If you are unable or unwilling to answer a question, I will of course respect that.*

- *First of all, I would like to inform you about the protection of your data:*

Data protection information:

- *We assure you that any information you provide will be treated in the strictest confidence. All legal requirements regarding data protection will be complied with. Personal data about you will be stored in accordance with data protection regulations and will be deleted after the analysis has been completed. You will not suffer any disadvantage if you do not participate. Your participation is completely voluntary. You may withdraw from the interview at any time without any penalty.*

- *Have you already signed the consent and privacy form before the project starts, but still have questions?*

<table>
<tr><th>Narrative stimulus</th><th>Checklist of research objects</th><th>Immanent/exmanent narrative enquiries</th><th>Paraphrasing/balancing</th></tr>
<tr><td>How has VaO affected your work (life?)?</td><td>U1: Changes due to the use of VaO in everyday working life</td><td>In which areas have there been significant changes?</td><td rowspan="2">Am I right in assuming that...
Overall, it can be judged that...
Is there anything else?
Can you describe it in more detail?
What do you mean by that?
What do you mean by that?
I have to ask again: XXXX
Can you go into more detail?</td></tr>
<tr><td>Please briefly explain how you handle the organization of the VaO room.</td><td>U1: Patient selection
- Patient selection
- Special aspects

U2: Appointment management
- Patient coordination
- Consideration of patient preference

U3: Study participation
- Compare effort for study participation and effort for room utilisation (e.g. Questionnaires)</td><td>U1: Patient selection
• Please tell me how you selected the people who come to the VaO room.Which aspects have a particular influence on the selection of VaO patients?
U2: Appointment management
• I am also interested in your appointment management. How do you assess your patient coordination since you also use the VaO room?
• Do you adapt your utilisation times to the needs of the patients?</td></tr>
</table>

Narrative stimulus	Checklist of research objects	Immanent/exmanent narrative enquiries	Paraphrasing/balancing
Please describe the differences between care in the VaO room and care at home.	U1: **Difference to care at home in the procedure of care**<br>- Efficiency<br>- Differences in patient contact	• What exactly makes the room more efficient than the care at home?<br>• Does the use of the room have an impact on waiting times for the VERAH and/or patient?<br>• To what extent does the use of the room have an influence on the working atmosphere (mood - colleagues, patients, boss)?	Am I right in assuming that...<br>Overall, it can be judged that...<br>Is there anything else?<br>Can you describe it in more detail?<br>What do you mean by that?<br>What do you mean by that?<br>I have to ask again: XXXX<br>Can you go into more detail?
Please describe to what extent the VaO room fulfils your needs in terms of patient care.	U1: **Equipment**<br>- Adequate?<br>- Missing equipment?	**U1: Equipment**<br>• What features of the rooms have helped you to provide your patients with adequate care?<br>• In your opinion, what would be missing in the VaO rooms to improve patient care?	

Narrative stimulus	Checklist of research objects	Immanent/exmanent narrative enquiries	Paraphrasing/balancing
Can you please describe to me what potential improvements you see for VaO?	U1: **Organisation**<br>- Negative aspects<br>- Potential for improvement<br><br>U2: **Patient care**<br>**- P**otential for improvement	**U1: Organisation**<br>• What specific aspects did you find problematic in the organisation of VaO?<br>• How can these aspects be improved so that the organisation runs smoothly?<br><br>**U2: Patient care**<br>• What potential for improvement do you see with regard to patient care?	Am I right in assuming that...<br>Overall, it can be judged that...<br>Is there anything else?<br>Can you describe it in more detail?<br>What do you mean by that?<br>What do you mean by that?<br>I have to ask again: XXXX<br>Can you go into more detail?

<table>
<tr><th>Narrative stimulus</th><th>Checklist of research objects</th><th>Immanent/exmanent narrative enquiries</th><th>Paraphrasing/balancing</th></tr>
<tr><td>How do you rate the relief VaO has provided to GPs?</td><td>U1: Workload reduction
- Recommendation

U2: Time saving
- Yes/no?
- How much?
- Why? Influencing factors</td><td>U1: Workload reduction
• To what extent would you recommend VaO to other practices to relieve the doctor's workload?
U2: Time saving
• Please tell me if using a VaO room saves time compared to traditional home visits?
• How much time do you estimate you save per day by using VaO?
• What factors influence this?</td><td rowspan="3">Am I right in assuming that...

Overall, it can be judged that...</td></tr>
<tr><td colspan="3">Now that we've talked a little bit about the VaO room, is there anything that we haven't mentioned that's important to you?</td></tr>
<tr><td colspan="3">How do you feel about VaO when you leave the focus group today?</td></tr>
</table>
